# Sonic Hedgehog potentiates BMP9-induced osteogenic differentiation of mesenchymal stem cells

**DOI:** 10.1016/j.gendis.2024.101308

**Published:** 2024-04-14

**Authors:** Lulu Zhang, Caixia Ji, Ziyun Li, Habu Jiwa, Zhou Xie, Xiaoji Luo, Jinyong Luo

**Affiliations:** a Key Laboratory of Diagnostic Medicine Designated by the Chinese Ministry of Education, Chongqing Medical University, Chongqing 40016, China; b Department of Clinical Laboratory, People's Hospital of Deyang City, Deyang, Sichuan 618000, China; c Department of Orthopedics, The First Affiliated Hospital of Chongqing Medical University, Chongqing 400016, China

**Keywords:** BMP9, Bone defects, Bone morphogenetic proteins, Mesenchymal stem cells, Osteogenic differentiation, Sonic Hedgehog

## Abstract

Bone morphogenetic protein 9 (BMP9) has remarkable potential to induce the differentiation of mesenchymal stem cells (MSCs) towards the osteoblastic lineage. Additionally, research suggests that certain growth factors have the ability to potentiate BMP9-induced osteogenic differentiation of MSCs. Sonic Hedgehog (Shh) plays an indispensable role in the regulation of skeletal development. The objective of this research was to assess the potential influence of Shh on BMP9-induced osteogenic differentiation of MSCs. Our findings indicated that Shh effectively enhanced BMP9-induced early and late osteogenic differentiation of MSCs, and increased BMP9-induced expression/transcriptional activity of osteogenesis-related transcription factors. Besides, it was observed that Shh promoted BMP9-induced ectopic bone formation of MSCs *in vivo*. Moreover, BMP9 was able to facilitate the repair of bone defects in rats, while Shh further accelerated this reparative process. Mechanistically, Shh enhanced the activation of the Smad1/5/8 signaling pathway which was induced by BMP9. Furthermore, GANT-61, an inhibitor of Gli1 and Gli2, attenuated the enhancing effect of Shh on BMP9-induced osteogenic differentiation of MSCs. Collectively, the co-administration of BMP9 and Shh may present a promising therapeutic approach for the treatment of fracture nonunion, delayed fracture healing, and bone defects.

## Introduction

Mesenchymal stem cells (MSCs) are a population of adult pluripotent cells characterized by robust self-renewing capacity and multi-directional differentiation potential.^1^ MSCs originate from the mesoderm and ubiquitously reside in bone marrow, dental pulp, placenta, fat, blood, umbilical cord, and other tissues.^1^^,^^2^ Upon exposure to specific stimuli *in vivo* and/or *in vitro*, MSCs can differentiate into osteoblasts, fibroblasts, odontoblasts, chondrocytes, muscle cells, and various other cell types.^1^^,^^2^ Due to their self-renewal potential, long-term *in vitro* proliferation ability, paracrine function, immunomodulation, and excellent safety profile, MSCs are considered highly advantageous for applications in cell therapy, tissue bioengineering, and regeneration medicine.^3^^,^^4^ In 1965, Urist first isolated and identified a group of growth factors derived from the bone extract and named bone morphogenetic proteins (BMPs) because of their strong ectopic osteo-inductive activity.^5^ Currently, BMPs are recognized as multifunctional morphogens that play pivotal roles in the regulation of embryogenesis, organ development, cell proliferation, and cell differentiation.^5^, ^6^, ^7^, ^8^, ^9^, ^10^ Within the skeletal system, BMPs directly affect the osteogenic differentiation of stem cells and are deeply involved in the development of bone tissue. Dysregulation or mutation of BMPs and/or their downstream signaling molecules may lead to various bone disorders such as BDA2 (brachydactyly type A2 and FOP (fibrodysplasia ossificans progressiva.^11^, ^12^, ^13^ It is important to note that not all BMPs exhibit osteo-inductive properties. To date, members of BMPs with characteristic osteo-inductive activity mainly include BMP2, BMP4, BMP6, and BMP7, of which BMP2 and BMP7 have been clinically used to treat bone fractures and bone nonunions.^14^, ^15^, ^16^, ^17^

As a relatively understudied member of BMPs, BMP9 (bone morphogenetic protein 9) is mainly synthesized and secreted by hepatic stellate cells.^18^ BMP9 has been proved to induce vascular maturation and angiogenesis,^19^ maintain cholinergic neuron phenotypes,^20^ promote lipid metabolism but inhibit liver glycogen production,^21^ regulate iron homeostasis,^22^ and control lymphatic vessel maturation and valve formation.^23^ Additionally, BMP9 is extensively involved in the pathogenesis of many diseases, including hereditary hemorrhagic telangiectasia,^24^ organ fibrosis,^25^ idio pathicpulmonary arterial hypertension,^26^ and malignant cancers.^27^, ^28^, ^29^, ^30^ Notably, recent studies have highlighted BMP9's potential to induce osteogenic differentiation of MSCs, even surpassing the efficacy of BMP2 and BMP7.^31^, ^32^, ^33^, ^34^, ^35^, ^36^, ^37^ Moreover, when combined with other growth factors such as IGF2, Leptin, and Wnt11, BMP9 exhibited more robust promoting effects on the osteogenic differentiation of MSCs. Hence, these combinations may present a promising strategy for promoting bone regeneration.^38^, ^39^, ^40^, ^41^

Sonic Hedgehog (Shh), a member of the Hedgehog (Hh) family, plays a vital role in various biological processes including tissue growth, tissue repair, organ development, organ regeneration, and maintenance of homeostasis.^42^^,^^43^ Within the skeletal system, Shh performs basic regulatory functions in vertebrate bone development, bone repair, and bone regeneration.^44^, ^45^, ^46^ Studies have shown that mice with silenced or deleted Shh genes can develop impaired trabecular bone formation, severe bone deformity, and delayed ossification.^44^, ^45^, ^46^ Previous research has indicated an important role of Shh in the osteogenic differentiation of MSCs.^44^, ^45^, ^46^ This study aimed to investigate whether Shh had the potential to enhance BMP9-induced osteogenic differentiation of MSCs. The results demonstrated that Shh effectively prompted BMP9-induced osteogenic differentiation of MSCs, and accelerated BMP9-promoted bone defect healing in rats. Mechanistic analysis revealed that Shh significantly augmented the activation of the Smad1/5/8 signaling pathway induced by BMP9 in MSCs. Furthermore, Gli1 (glioma-associated oncogene 1) and Gli2 (glioma-associated oncogene 2), the key transcription factors of Shh signaling, most likely mediated the augmentative effects of Shh on BMP9-induced osteogenic differentiation. Overall, this study suggests that the combination of BMP9 and Shh may achieve more potent osteo-inductive effects in MSCs, offering a promising strategy to improve the therapeutic outcomes of some challenging bone problems, including fracture nonunion, delayed fracture healing, and bone defects.

## Materials and methods

### Cell culture and chemical reagents

MSC cell lines (C3H10T1/2 and C2C12) and HEK-293 cell line were obtained from ATCC (Manassas, VA, USA). Mouse embryonic fibroblasts were isolated from fetal mice on post-coitus day 13.5 according to previously reported protocols.^47^ All cells were cultured in a complete DMEM medium containing 10% fetal bovine serum. Gli1/Gli2 inhibitor (GANT-61) was purchased from Selleck (Houston, TX, USA).

### Recombinant adenovirus construction

The recombinant adenovirus expressing BMP9 was constructed in our previous research and named Ad-BMP9.^48^ In the present study, the adenovirus expressing exogenous mouse Shh was constructed using the Adeasy system according to the described standard construction protocols.^48^

### ALP activity detection

ALP (alkaline phosphatase) activity was visualized by cytochemical staining assay, which used Fast Blue BB salt napthol and AS-MX phosphate as substrates to generate positive staining, as described in our previous report.^47^ For ALP quantitative detection, MSCs were lysed with cell lysis buffer. Subsequently, cell lysates were centrifuged (1000× *g*, 2 min) to obtain the supernatants. Then, ALP activity was quantitatively determined according to the protocol established in our previous study.^47^

### Assessment of matrix mineralization

First, Alizarin red S dye was prepared into 0.4% working solution (pH 8.0). After fixation with 0.4% glutaraldehyde, cells in 24-well plates were incubated with 0.4% working solution. When visible red calcium nodules were observed under the microscope, the 0.4% working solution was completely discarded. Next, 200 μL of distilled water was added to totally cease the staining process. Finally, the red matrix mineralization in cells was observed and photographed under a light microscope.

### RNA extraction and quantitative PCR

Total cellular RNA was purified using Trizol RNA Extraction Reagent according to the protocol established in our previous study.^47^ To generate cDNA templates, the RNA was subjected to a reverse transcription reaction, following the previously described protocol.^47^ Quantitative PCR was performed according to the user manual of SYBR Green PCR Master Mix, as reported previously.^47^ The internal reference gene GAPDH was included for normalization. The 2^−ΔΔCt^ assay was utilized to determine the relative mRNA expression levels.

### Western blot

Cells were lyzed with Western/IP cell lysis buffer, followed by centrifugation at 13,000× *g* to obtain supernatants. The collected supernatants were boiled to denature protein, and subjected to protein concentration determination by BCA assay. Next, the samples were electrophoresed on 10% SDS-PAGE gel and transferred onto PVDF membranes. After incubation with 5% BSA solution to prevent non-specific binding, the membranes were incubated with 1:1000 diluted primary antibodies at 4 °C overnight. Then, the membranes were rinsed three times with 1 × TBST, followed by a reaction with secondary antibodies conjugated with horseradish peroxidase at a 1:1000 dilution. The target protein in PVDF membranes was visualized using an enhanced chemiluminescence kit and then photographed using a gel imaging system.

### Luciferase reporter assay

MSCs were cultured in T25 flasks until cell confluence reached 50%∼60%. Next, the luciferase reporter plasmid p6 × OSE-luc or p12 × SBE-luc was transfected into MSCs with Lipofectamine™ 3000 according to the standard user protocol. MSCs were cultured for another 16 h and then seeded into 24-well plates (5 × 10^4^ cells/well). Subsequently, cells were infected with the indicated adenovirus for 24 h or 36 h and were subjected to luciferase activity determination according to the previously described protocol.^47^

### Subcutaneous MSC implantation model

Approval for all animal experiments conducted in this present study was granted by the Ethics Committee of Chongqing Medical University. Female BALB/c nude mice (28 days old, weighing 20 ± 2 g) were obtained from Beijing Huafukang Biotechnology Co. LTD. Animals were cared for and handled according to the standard protocol. MSCs (C3H10T1/2 and C2C12) were cultured in T75 flasks until cell confluence reached 60%. Cells were then infected with the designated adenovirus and cultured for an additional 24 h. After that, cells were harvested and then injected subcutaneously into the flanks of nude mice at a dose of 5 × 10^6^ cells per injection. The experimental grouping was as follows: Blank, BMP9, Shh, and Shh + BMP9. At 5 weeks after model establishment, all nude mice were humanely euthanized for micro-CT scanning, osteometric analysis, and histological staining analysis.

### Live/dead cell staining

A calcein-AM/PI double staining kit (YEASON, China) was used to evaluate the viable (green calcein staining) and dead (red PI staining) cells. In the BMSCs (bone marrow mesenchymal stem cells) group, rat BMSCs were seeded into 96 well plates at a density of 1 × 10^4^ cells per well. In the BMSCs+5% GelMA group, rat BMSCs (1 × 10^4^ cells) were mixed with 10 μL of 5% GelMA and then seeded into 96 well plates. Next, cells were stained with Calcein-AM/PI working solution containing PI (4.5 μM) and calcein-AM (2 μM) at 37 °C for 15 min at the indicated time point. After washing with phosphate buffer saline solution 3 times, cells were observed with a fluorescence microscope.

### Rat cranial defect repair model

Rat BMSCs were isolated as described previously,^49^ and then subjected to surface marker detection by flow cytometry. After anesthetization and exposure of the cranium, a critical-sized bone defect with a diameter of 5 mm was made on the right side of the skull using a dental trephine drill. Next, BMSCs (3 × 10^6^ cells) were infected with the indicated adenovirus and cultured for an additional 24 h. Thereafter, cells were harvested and mixed with 20 μL of 5% GelMA solution. Subsequently, the GelMA/cell mixtures were injected into calvarial defect sites and then hardened by 10 s of exposure to ultraviolet light. The experimental grouping was as follows: Blank, 5% GelMA, 5% GelMA + BMSCs, 5% GelMA + BMP9, 5% GelMA + Shh, and 5% GelMA + Shh + BMP9. At 45 days after model establishment, all rats were humanely euthanized for micro-CT scanning and histological staining analysis.

### Statistical analysis

To conduct statistical analysis, the quantitative assays were carried out in triplicate, and the results were replicated in a minimum of three independent experiments. Quantitative data were represented as mean ± standard deviation. The One-way ANOVA or student's *t*-test was utilized to compare the differences between two treatment groups. *p* values less than 0.05 were considered statistically significant and *p* values less than0.01 were considered notably statistically significant.

## Results

### Shh enhances BMP9-induced early and late osteogenic differentiation of MSCs

**Figure 1:**
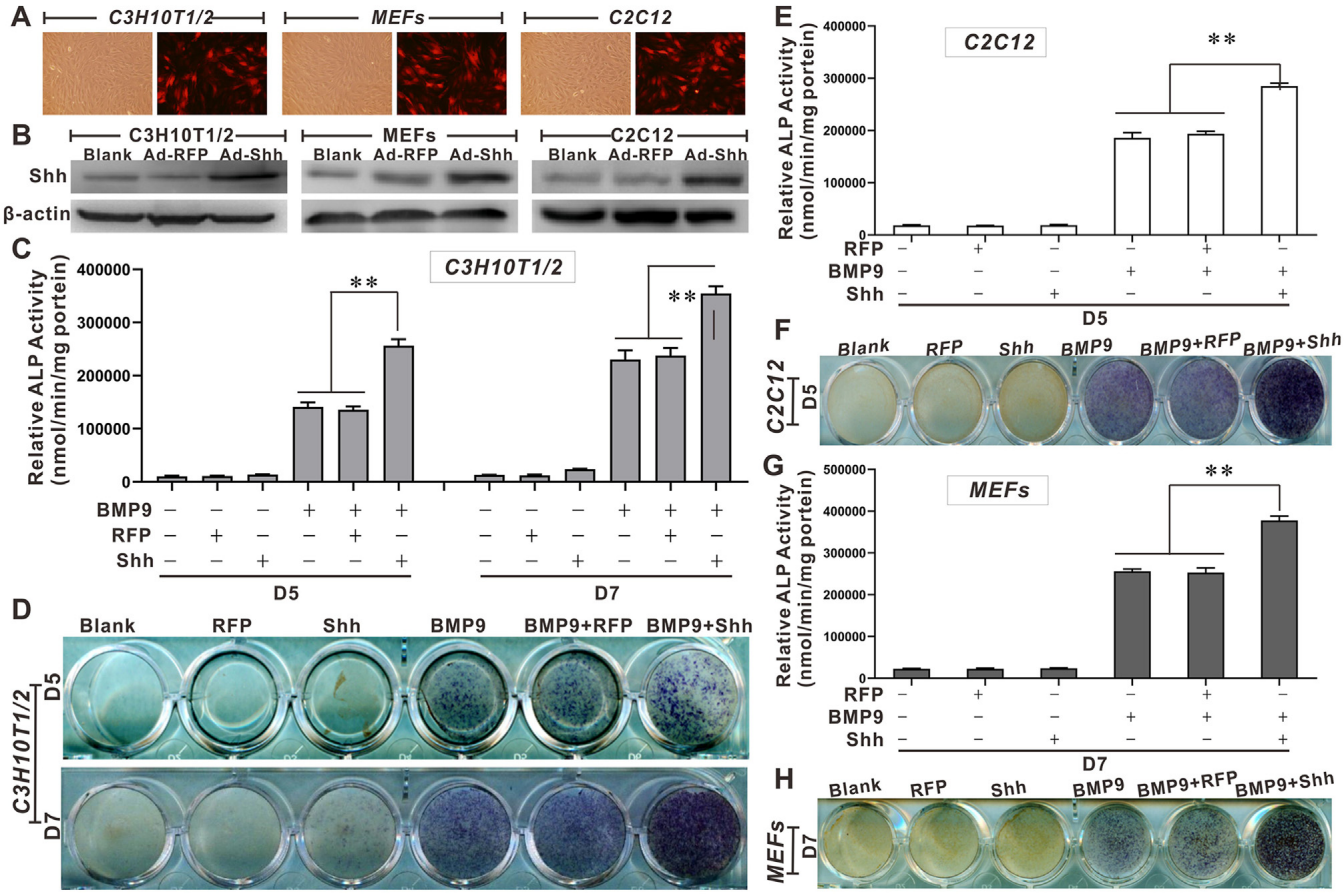
The effects of Shh on BMP9-induced early osteogenic differentiation of MSCs. **(A)** The infection of Ad-Shh in MSCs (fluorescence microscope, × 100). **(B)** Validation of the expression of Shh by Ad-Shh in MSCs (Western blot). **(C, D)** The effects of Shh on BMP9-induced ALP activity in C3H10T1/2 cells (C: chemiluminescence; D: cytochemical staining). **(E, F)** The effects of Shh on BMP9-induced ALP activity in C2C12 cells (E: chemiluminescence; F: cytochemical staining). **(G, H)** The effects of Shh on BMP9-induced ALP activity in MEFs (G: chemiluminescence; H: cytochemical staining). ∗*p* < 0.05, ∗∗*p* < 0.01; ALP, alkaline phosphatase; BMP9, bone morphogenetic protein 9; MSCs, mesenchymal stem cells; Shh, Sonic Hedgehog.

**Figure 2:**
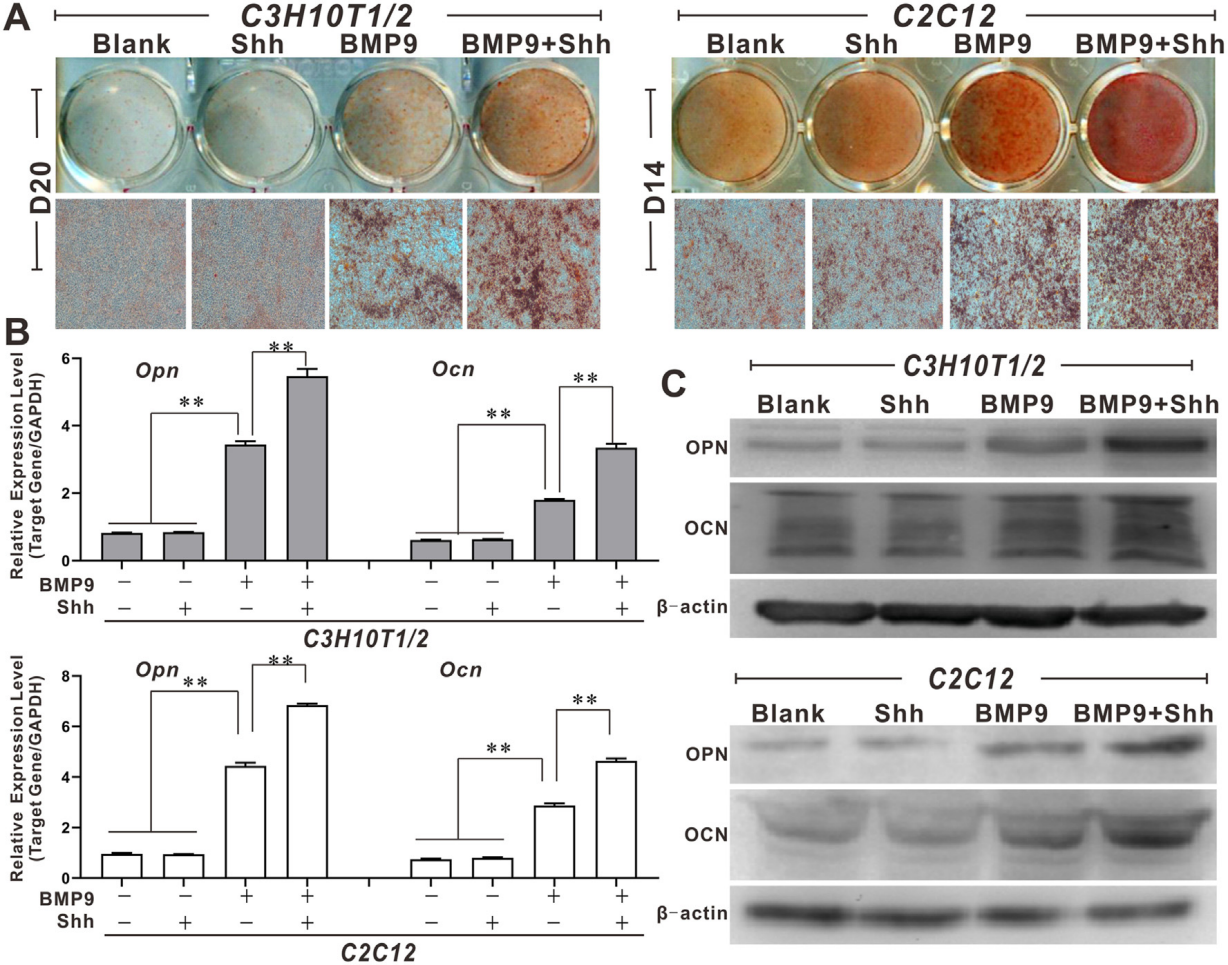
The effects of Shh on BMP9-induced late osteogenic differentiation of MSCs. **(A)** The effects of Shh on BMP9-induced matrix mineralization of MSCs (alizarin red S staining, × 150). **(B)** The effects of Shh on BMP9-induced mRNA levels of OPN and OCN in MSCs (quantitative PCR). **(C)** The effects of Shh on BMP9-induced protein levels of OPN and OCN in MSCs (Western blot). ∗*p* < 0.05, ∗∗*p* < 0.01. BMP9, bone morphogenetic protein 9; MSCs, mesenchymal stem cells; OCN, osteocalcin; OPN, osteopontin; Shh, Sonic Hedgehog.

First, we validated that the adenovirus expressing exogenous mouse Shh was able to effectively infect MSCs (Fig. 1A) and obviously increased the Shh protein level in MSCs (Fig. 1B). Then, we decided to investigate whether Shh had any influence on BMP9-induced osteogenic differentiation of MSCs. We found that although Shh itself had no visible effect on ALP, a commonly used indicator of early osteogenic differentiation, it significantly promoted BMP9-induced ALP activity in C3H10T1/2 cells (Fig. 1C, D). Moreover, we also obtained similar results of ALP activity in C2C12 cells (Fig. 1E, F) and mouse embryonic fibroblasts (Fig. 1G, H), which are also common sources of MSCs.^39^, ^40^, ^41^^,^^47^ These results strongly suggest that Shh may further promote BMP9-induced early osteogenic differentiation of MSCs. Next, we investigated whether Shh affected BMP9-induced late osteogenic differentiation of MSCs by detecting the mineralized matrix, a recognized marker of late osteogenic differentiation. Compared with cells treated with BMP9 alone, we observed significantly more red nodules in BMP9/Shh co-treated MSCs, indicating that Shh may augment BMP9-induced matrix mineralization of MSCs (Fig. 2A). In addition, we performed quantitative PCR and Western blot assays, and the results demonstrated that BMP9-induced mRNA and protein levels of OPN (osteopontin) and OCN (osteocalcin) in MSCs were effectively elevated by Shh (Fig. 2B, C). Taken together, these results strongly suggest that Shh may promote BMP9-induced early and late osteogenic differentiation of MSCs, although Shh alone shows no substantial osteo-inductive effects on MSCs.

### Shh promotes BMP9-induced expression/transcriptional activity of osteogenesis-related transcription factors in MSCs

**Figure 3:**
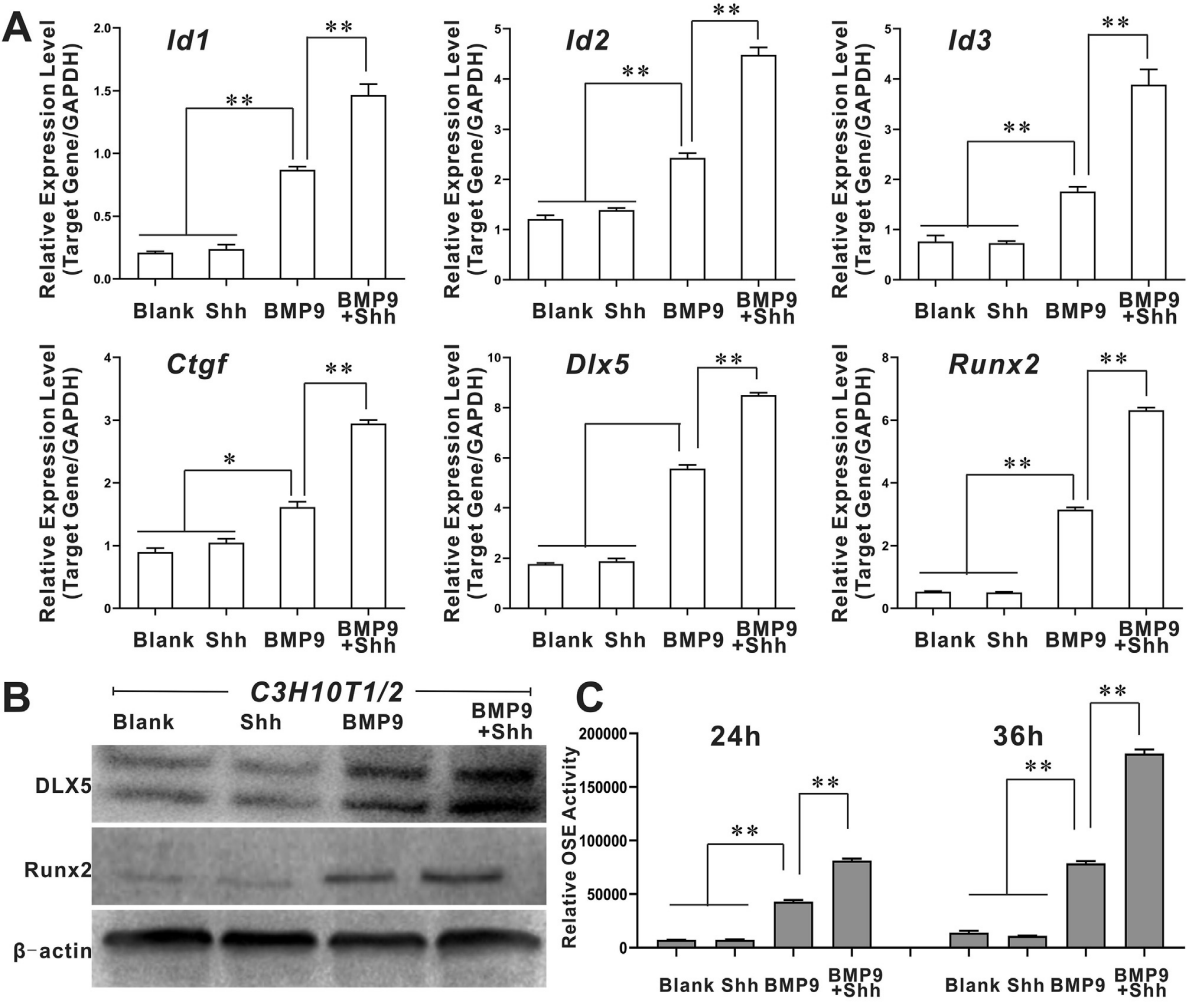
The effects of Shh on BMP9-induced expression/activity of pivotal osteogenic transcriptional factors in MSCs. **(A)** The effects of Shh on the expression of Id1, Id2, Id3, CTGF, Runx2, and DLX5 in C3H10T1/2 cells (quantitative PCR). **(B)** The effects of Shh on the expression of Runx2 and DLX5 in C3H10T1/2 cells (Western blot). **(C)** The effects of Shh on BMP9-induced Runx2 transcriptional activity in C3H10T1/2 cells (Luciferase reporter assay). ∗*p* < 0.05, ∗∗*p* < 0.01. BMP9, bone morphogenetic protein 9; CTGF, connective tissue growth factor; DLX5, distal-less homeobox 5; Id1/2/3, inhibitor of DNA binding 1/2/3; MSCs, mesenchymal stem cells; Runx2, Runt-related transcription factor 2; Shh, Sonic Hedgehog.

Transcription factors, including Runx2 (Runt-related transcription factor 2), CTGF (connective tissue growth factor), Dlx5 (distal-less homeobox 5), and inhibitors of DNA binding proteins (Id1, Id2, Id3), have been proven to be the osteogenesis-related targets of BMP9.^34^^,^^50^, ^51^, ^52^ Using quantitative PCR, we demonstrated that BMP9-induced mRNA levels of Id1, Id2, Id3, CTGF, Runx2, and Dlx5 were increased by Shh (Fig. 3A). Western blot confirmed that Dlx5 and Runx2 protein levels in BMP9/Shh co-stimulated MSCs were elevated compared with those in MSCs treated with BMP9 alone (Fig. 3B). Next, we decided to verify whether BMP9-induced Runx2 transcriptional activity was also enhanced by Shh as well. The luciferase reporter plasmid p6 × OSE-luc contains six consecutive OSE elements that are controlled by Runx2, and represent the transcriptional activity of Runx2.^47^^,^^53^ Accordingly, we found that the p6 × OSE-luc luciferase activity was further increased in BMP9/Shh co-treatment group compared with the group treated with BMP9 alone (Fig. 3C), thus suggesting that Shh may enhance BMP9-induced transcriptional activity of Runx2. Collectively, these results indicate that Shh can promote BMP9-induced expression of osteogenesis-related transcription factors, and enhance the transcriptional activity of Runx2 in MSCs.

### Shh enhances BMP9-induced ectopic bone formation of MSCs

**Figure 4:**
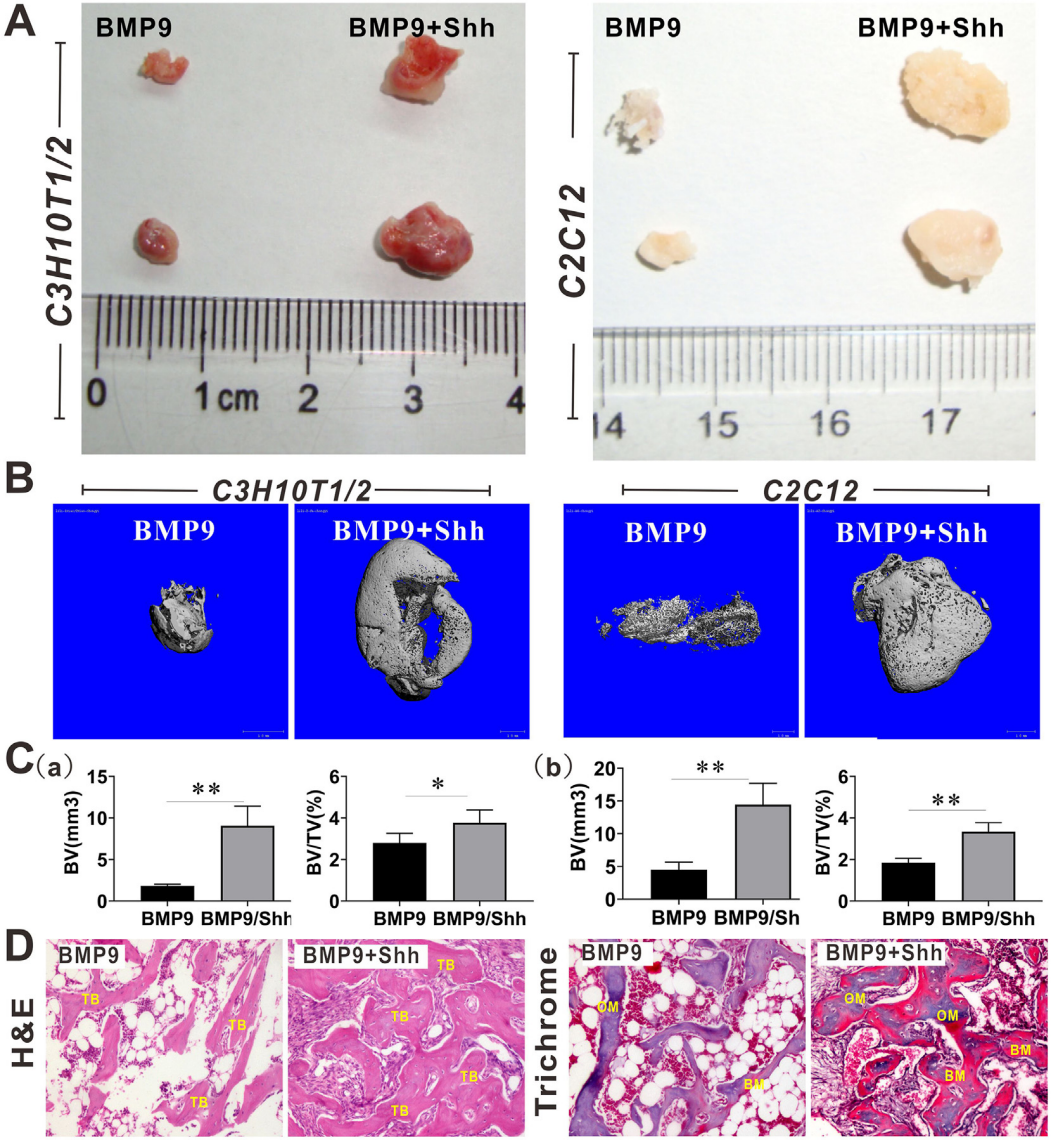
The effects of Shh on BMP9-induced ectopic bone formation of MSCs. **(A)** Representative bone masses from the ectopic bone formation induced by BMP9 alone or BMP9/Shh of C3H10T1/2 cells and C2C12 cells. **(B)** 3D reconstruction of bone masses by micro-CT analysis. **(C)** BV and BV/TV results of bone masses. (a) The results of bone masses formed by C3H10T1/2 cells. (b) The results of bone masses formed by C2C12 cells. **(D)** Histologic evaluation of retrieved bone masses ( × 200). ∗*p* < 0.05, ∗∗*p* < 0.01. BMP9, bone morphogenetic protein 9; BV, bone volume; CM, chondroid matrix; OM, osteoid matrix; Shh, Sonic Hedgehog; TB, trabecular bone; TV, total tissue volume.

Next, we established a subcutaneous MSC implantation model to verify the enhancing effects of Shh on BMP9-induced osteogenic differentiation of MSCs *in vivo*. Blank MSCs and Shh-treated MSCs failed to form any visible subcutaneous masses in nude mice. As shown in Figure 4A, both BMP9-treated MSCs and BMP9/Shh-co-treated MSCs formed obvious subcutaneous masses. More importantly, the subcutaneous masses formed by BMP9/Shh-co-treated cells appeared to be apparently larger than those formed by BMP9-treated cells (Fig. 4A), which was also supported by the results of the micro-CT scan as well (Fig. 4B). Furthermore, the quantitative osteometric analysis of micro-CT showed that the bone volume was much larger, and bone volume fraction (bone volume/total tissue volume) was significantly higher when BMP9 was combined with Shh (Fig. 4C). Hematoxylin-eosin staining analysis of the retrieved subcutaneous masses showed that BMP9/Shh combination treatment produced more trabeculae than BMP9 alone (Fig. 4D). In addition, Masson's trichrome staining showed a greater presence of bone matrix in samples harvested from the BMP9/Shh combination group (Fig. 4D). Thus, these results may provide preliminary evidence to confirm the promoting effects of Shh on BMP9-induced osteogenic differentiation *in vivo*.

### Shh accelerates BMP9-induced bone defect repair in rats

**Figure 5:**
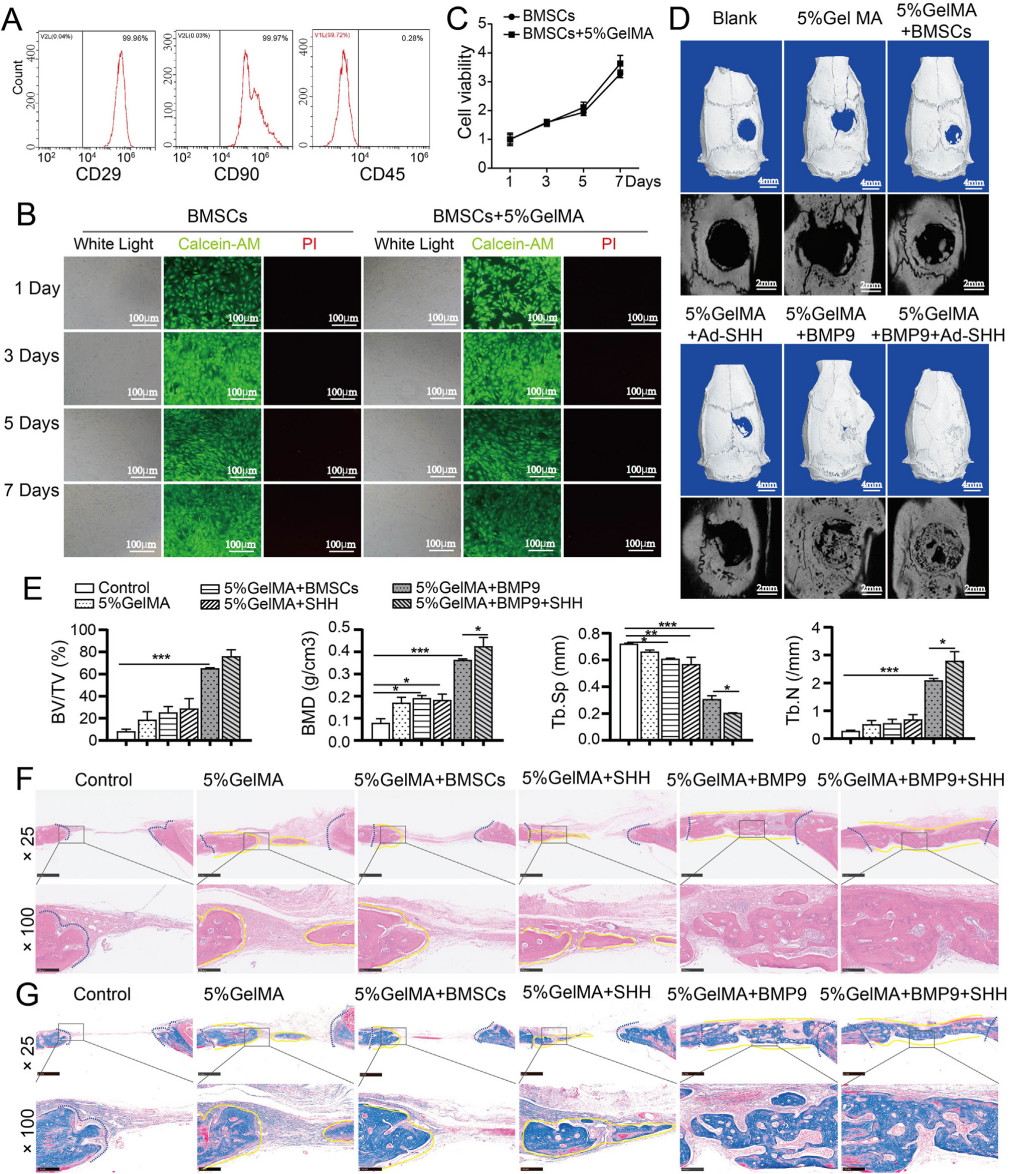
The effects of Shh on BMP9-induced bone defect repair in rats. **(A)** The identification of surface markers of rat BMSCs. **(B)** The effects of GeLMA on rat BMSC viability (calcein/PI staining). **(C)** The effects of GeLMA on rat BMSC viability (MTT assay). **(D)** The effects of Shh on BMP9-induced bone defect repair (micro-CT). **(E)** The quantified data of the micro-CT analysis. **(F)** Histologic evaluation of retrieved bone masses (hematoxylin-eosin staining). Blue dashed lines indicate the edges of the original bone defect and newly bone areas are outlined with yellow solid lines. **(G)** Histologic evaluation of retrieved bone masses (Mason's staining). Blue dashed lines indicate the edges of the original bone defect and newly bone areas are outlined with yellow solid lines. ∗*p* < 0.05, ∗∗*p* < 0.01. BMP9, bone morphogenetic protein 9; BMSC, bone marrow mesenchymal stem cell; Shh, Sonic Hedgehog.

We then established a bone defect repair model in rats to investigate whether Shh accelerated BMP9-induced bone regeneration. We isolated BMSCs from rat bone marrow and demonstrated that the surface protein markers on the isolated cells were CD45^−^, CD90^+^, and CD29^+^ (Fig. 5A), indicating that the isolated cells were BMSCs. Next, we found that the PI-stained dead cells were almost invisible in BMSCs mixed with 5% GelMA (Fig. 5B), suggesting that the viability of BMSCs may not be affected by 5% GelMA. The results of the MTT assay further confirmed that 5% GelMA had no perceptible effect on the viability of BMSCs. Therefore, this may indicate that GelMA has relatively good biocompatibility. We then surgically created a critical-sized cranial defect and validated using a micro-CT scan that BMP9 evidently induced bone regeneration in the cranial defect area (Fig. 5D, E). It is worth noting that Shh further enhanced the bone regeneration induced by BMP9 (Fig. 5D), which was manifested as an increase in bone mineral density and trabecular number, while a decrease in trabecular spacing (Fig. 5E). Histological analysis showed that BMP9 in combination with Shh induced more sheet-like trabecular bone formation in the cranial defect area (Fig. 5F) and produced a much more bone-like matrix (Fig. 5G). These results suggest that BMP9 can promote bone defect repair in rats and Shh can notably accelerate this repair process.

### Shh promotes BMP9-triggered activation of Smad signaling pathway in MSCs

**Figure 6:**
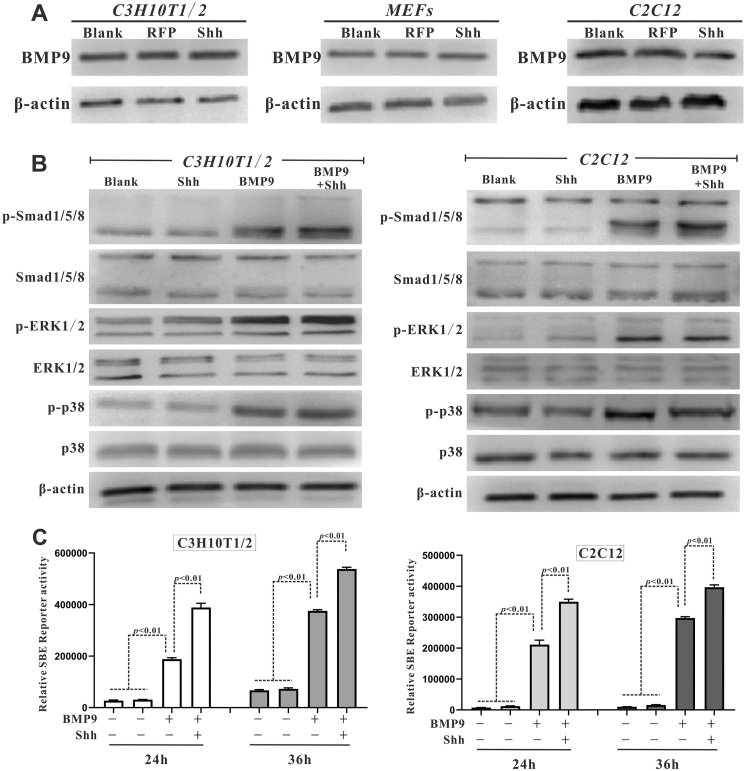
The effects of Shh on BMP9-induced activation of Smad1/5/8 and MAPK signaling in MSCs. **(A)** The effects of Shh on BMP9 expression in MSCs (Western blot). **(B)** The effects of Shh on BMP9-induced activation of Smad1/5/8 and MAPK signaling in C3H10T1/2 cells and C2C12 cells (Western blot). **(C)** The effects of Shh on BMP9-induced Samd1/5/8 transcriptional activity in C3H10T1/2 cells and C2C12 cells (luciferase reporter assay). ∗*p* < 0.05, ∗∗*p* < 0.01. BMP9, bone morphogenetic protein 9; MAPK, mitogen-activated protein kinase; MSCs, mesenchymal stem cells; Shh, Sonic Hedgehog.

Next, we thought that the molecular mechanisms by which Shh enhanced BMP9-induced osteogenic differentiation of MSCs ought to be preliminarily analyzed. Using Western blot analysis, we demonstrated that Shh did not alter the exogenous protein level of BMP9 in MSCs (Fig. 6A). Smad1/5/8, ERK1/2 (extracellular signal-regulated kinase 1/2), and p38 have been shown to be downstream signaling pathways that mediate the osteo-inductive effects of BMP9 in MSCs.^32^ In this study, the results of Western blot revealed that although BMP9 stimulated ERK1/2 and p38 phosphorylation, Shh did not influence the increase in ERK1/2 and p38 phosphorylation induced by BMP9 in MSCs (Fig. 6B). BMP9 also increased Smad1/5/8 phosphorylation, and this effect was notably enhanced by the presence of Shh (Fig. 6B), indicating that Shh may promote BMP9-induced Smad1/5/8 phosphorylation. The luciferase reporter plasmid p12 × SBE-luc contains twelve consecutive BMP-responsive elements and indicates the transcriptional activity of Smad1/5/8.^47^ Here, we validated that Shh enhanced BMP9-induced transcriptional activity of Smad1/5/8, resulting in a further increased luciferase activity of p12 × SBE-luc (Fig. 6C). To sum up, our results suggest that Shh can potentiate the activation of canonical Smad1/5/8 signaling pathway induced by BMP9.

Transcription factors Gli1 and Gli2 are likely to mediate the stimulatory effect of Shh on BMP9-induced osteogenic differentiation.

**Figure 7:**
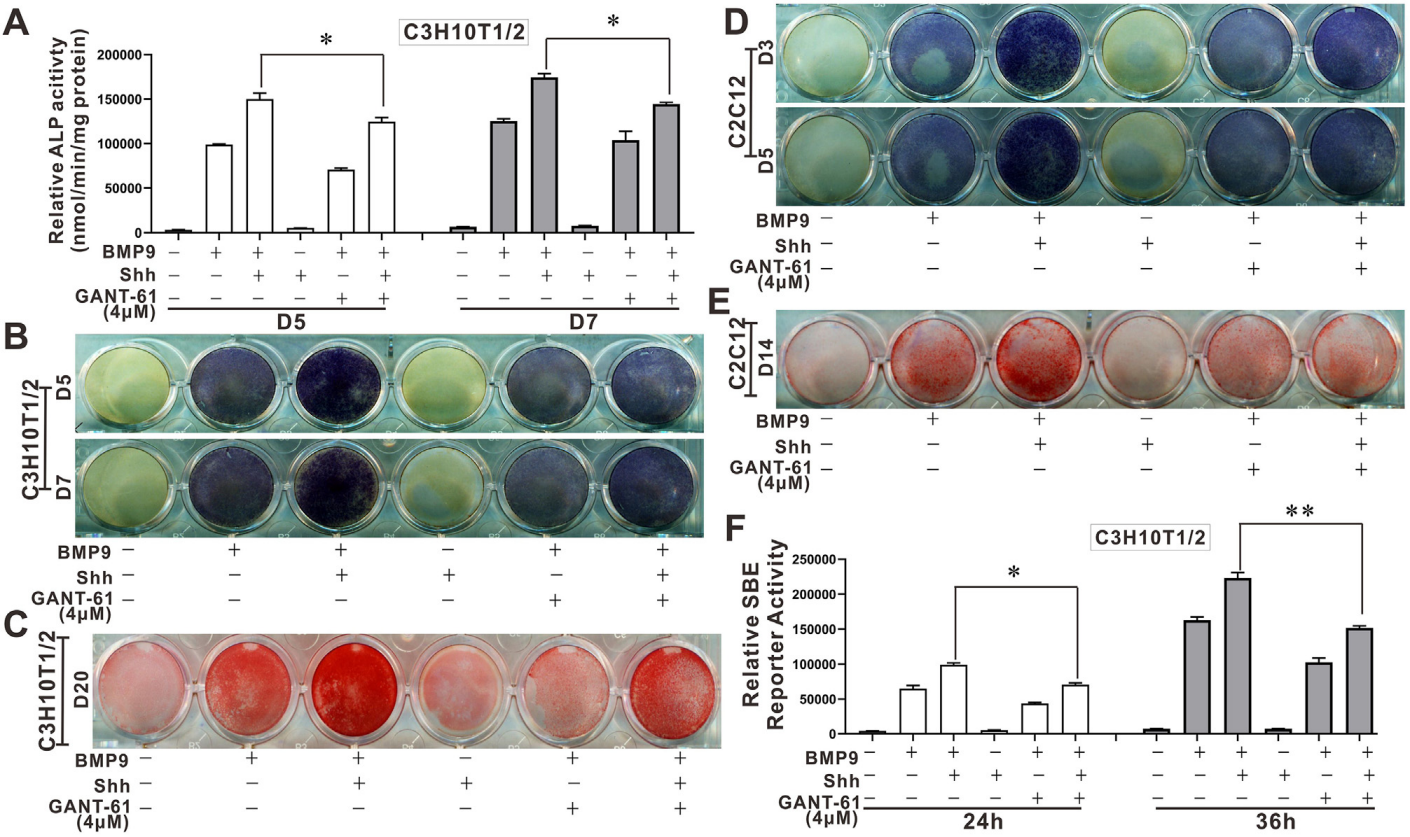
The effects of Gli1/Gli2 inhibitor GANT-61 on the enhancing impacts of Shh on BMP9-induced osteogenic differentiation of MSCs. **(A)** The effects of GANT-61 on the enhancing effects of Shh on BMP9-induced ALP activity in C3H10T1/2 cells (chemiluminescence). **(B)** The effects of GANT-61 on the enhancing effects of Shh on BMP9-induced ALP activity in C3H10T1/2 cells (cytochemical staining). **(C)** The effects of GANT-61 on the enhancing effects of Shh on BMP9-induced matrix mineralization in C3H10T1/2 cells (alizarin red S staining). **(D)** The effects of GANT-61 on the enhancing effects of Shh on BMP9-induced ALP activity in C2C12 cells (cytochemical staining). **(E)** The effects of GANT-61 on the enhancing effects of Shh on BMP9-induced matrix mineralization in C2C12 cells (alizarin red S staining). **(F)** The effects of GANT-61 on the enhancing effects of Shh on BMP9-induced Samd1/5/8 transcriptional activity in C3H10T1/2 cells (luciferase reporter assay). ∗*p* < 0.05, ∗∗*p* < 0.01. ALP, alkaline phosphatase; BMP9, bone morphogenetic protein 9; Gli1/2, glioma-associated oncogene 1/2; MSCs, mesenchymal stem cells; Shh, Sonic Hedgehog.

Transcription factors Gli1 and Gli2 are essential mediators in Shh signal transduction.^42^^,^^54^^,^^55^ Our recent study has proven that BMP9 increased the mRNA levels of Gli1 and Gli2.^56^ Therefore, we sought to analyze whether Gli1 and Gli2 were likely to be involved in the enhancing effect of Shh on BMP9-induced osteogenic differentiation of MSCs. We demonstrated that GANT-61 (4 μM), a specific inhibitor of Gli1 and Gli2, partially reversed the stimulatory effect of Shh on BMP9-induced ALP activity and matrix mineralization in C3H10T1/2 cells (Fig. 7A–C). Furthermore, we obtained similar results in C2C12 cells (Fig. 7D, E). In addition, although Shh promoted BMP9-induced transcription activity of Smad1/5/8, GANT-61 was found to abolish this enhancing phenotype, leading to a decrease in the transcription activity of Smad1/5/8 (Fig. 7F). Thus, transcriptional factors Gli1 and Gli2 are likely to mediate the stimulatory effect of Shh on BMP9-induced osteogenic differentiation of MSCs.

## Discussion

BMP9, compared with BMP2 and BMP7, exhibits stronger potential to stimulate the differentiation of MSCs into the osteoblastic lineage and may even be the most osteo-inductive member of the BMP family.^31^^,^^33^, ^34^, ^35^, ^36^, ^37^ In addition, when combined with other growth factors, BMP9 produces a more robust osteo-inductive effect, which may provide a novel strategy for enhancing bone regeneration.^38^, ^39^, ^40^, ^41^ In this study, we found that Shh alone did not show any obvious effect on the osteogenic differentiation of MSCs. However, it should be especially emphasized that Shh significantly promoted BMP9-induced osteogenic differentiation of MSCs, leading to a further increase in ALP activity, matrix mineralization, and the expression/activation of osteogenesis-related transcription factors. Moreover, Shh enhanced BMP9-induced ectopic bone formation by MSCs in nude mice. Although Shh itself did not noticeably contribute to the healing of bone defects in rats, it significantly accelerated the bone defect repair process promoted by BMP9. Mechanistically, we confirmed that the BMP9-induced activation of the Smad/1/5/8 signaling pathway was further enhanced by Shh. Furthermore, the enhancing effect of Shh on BMP9-induced osteogenic differentiation of MSCs was partially abolished by Gli1/Gli2 inhibitor GANT-61, implying a regulatory role of Gli1 and Gli2 involved.

The Hh signaling pathway is highly evolutionarily conserved in both invertebrates and vertebrates. It plays an essential role in embryonic development, organ size, tissue homeostasis, and cell differentiation.^42^^,^^54^^,^^55^ When the Hh ligand protein is at a low level or absent, Ptch (Patched) binds to Smo (Smoothened) and suppresses Smo activity, thereby maintaining the inactive state of the Hh signaling pathway. On the contrary, when the Hh protein binds to Ptch1, it counteracts the inhibitory effect of Ptch1 on Smo, resulting in the initiation of the Hh signaling pathway. The transduction of the Hh signaling pathway involves many downstream signal molecules, among which the Gli family of transcription factors is the most important mediator that binds to promoters and regulates target gene transcription.^42^ The Hh signaling pathway is a pivotal pathway that tightly controls the size of many organs, including the lung, the nerve, the limb, and the gastrointestinal tract.^42^^,^^54^^,^^55^^,^^57^ Meanwhile, it plays a significant role in the initiation and progression of certain tumors. The Hh signaling pathway is frequently over-activated in breast cancer, bladder cancer, digestive tract tumors, and neurocytomas.^58^^,^^59^ In addition, numerous studies have suggested that the Hh signaling pathway contributes indispensably to bone development and bone metabolism.^60^, ^61^, ^62^ Hh can not only directly regulate the formation of vertebrate bone but also control the osseous differentiation and cartilaginous differentiation.^60^ Moreover, Hh is one of the most important regulators of osteoblasts, which directly promotes the osteogenic differentiation of MSCs,^63^, ^64^, ^65^, ^66^, ^67^ and determines the cell fate of osteoblasts.^68^

As a member of the Hh family, Shh is expressed ubiquitously in most tissues. Dysfunction of Shh signaling may lead to related skeletal diseases, such as Smith-Lemli-Opitz syndrome.^69^ In Shh knockout mice, the development of vertebrae and distal bone was severely impaired,^60^, ^61^, ^62^^,^^69^ implying an essential role of Shh in skeletal development. In rat bone marrow-derived MSCs, Shh not only promoted ALP activity and matrix mineralization but also increased the expression of Runx2, OCN, and ColI (collagen type I).^63^ In C3H10T1/2 and MC3T3-E1 (a common pre-osteoblast cell line), Shh induced the expression of ALP and OCN.^67^^,^^69^ Interestingly, chicken fibroblasts transfected with Shh were demonstrated to effectively promote the osteogenic differentiation of MSCs and pro-osteoblasts in a co-culture system, and significantly induced intramuscular ectopic bone formation in male athymic mice.^70^ However, in a study using dental apical papilla MSCs, Shh was found to inhibit osteogenic differentiation by acting as a negative regulator of BMPs.^71^ Thus, Shh plays essential roles in the skeletal formation of vertebrates although its precise role in different cells/conditions remains to be deeply explored.

Id1, Id2, Id3, CTGF, Dlx5, and Runx2, are downstream transcription factors of BMP9 and play key roles in BMP9-induced osteogenesis.^34^^,^^50^, ^51^, ^52^ It has been reported that these osteogenesis-related transcription factors also participate in Shh-regulated osteogenesis and bone formation.^72^^,^^73^ For example, the gene knockout of Gli3, a well-established effector of Shh, resulted in abnormal expression of Runx2 and Dlx5 in calvarial mesenchyme, and premature fusion of cranial suture. However, down-regulation of Runx2 effectively prevented premature closure of cranial suture caused by Gli3 deletion.^72^ Notably, recombinant Shh protein was able to activate Runx2 through which to at least partially restore the osteogenic potency of BMSCs in high glucose conditions.^73^ A study by Tian et al showed that Shh effectively induced the differentiation of MC3T3-E1 pre-osteoblasts into osteoblasts. However, gene silencing of Osx (Osterix) blocked this osteo-inductive effect of Shh, indicating the necessary role of Osx in Shh-induced osteogenic differentiation.^74^ In the present study, we demonstrated that Shh further promoted the expression of these osteogenesis-related transcription factors induced by BMP9, and enhanced BMP9-induced transcriptional activity of Runx2. These phenomena strongly suggest that Shh has the potential to augment BMP9-induced osteogenic differentiation of MSCs by directly affecting the expression/activation of essential osteogenesis-related transcription factors.

Our previous studies have confirmed that BMP9 can activate Smad1/5/8, ERK1/2, and p38 signaling pathways.^32^^,^^47^ Blockage of Smad1/5/8 and p38 signaling pathways attenuated the osteogenic differentiation of MSCs induced by BMP9, whereas ERK1/2 inhibition led to an opposite effect, implying that these three signaling pathways had distinct regulatory roles in the osteo-inductive activity of BMP9.^32^^,^^47^ In this study, we discovered that Shh did not significantly influence ERK1/2 and p38 signaling pathways, as the phosphorylation levels of ERK1/2 and p38 remained unchanged following Shh treatment. It should be especially noted that Shh significantly enhanced BMP9-induced phosphorylation of Smad1/5/8 in MSCs. Furthermore, our results revealed that Shh increased the transcriptional activity of Smad1/5/8 induced by BMP9, indicating that Shh may further augment the BMP9-induced activation of the Smad1/5/8 signaling pathway. Previous studies have repeatedly demonstrated that BMP9 can activate TGFβ type Ⅰ receptors (activin receptor-like kinase 1/2) through which to directly phosphorylate Smad1/5/8.^47^^,^^75^^,^^76^ Here, we summarized at least two presumptive reasons for the enhancing effects of Shh on the BMP9-induced activation of the Smad1/5/8 signaling pathway. Firstly, Shh may directly affect the catalytic activity of TGFβ type Ⅰ receptors on Smad1/5/8 phosphorylation. Secondly, the downstream effectors of Shh, such as the Gli transcription factor family, may potentially increase the transcriptional regulatory activity of Smad1/5/8 by interacting with Smad1/5/8. However, further experiments are needed to fully clarify these issues.

The Gli family of transcription factors, comprising Gli, Gli2, and Gli3, serves as the principal regulator of the Hh signaling pathway.^42^ Although all three Gli transcription factors have strong DNA binding capabilities, they differ structurally, mainly because the Gli1 protein lacks an N-terminal inhibitory region.^77^^,^^78^ More importantly, Gli proteins not only have structural differences but also display distinct functions. Gli1 is a well-established transcription activator, Gli3 is a recognized transcription repressor, while Gli2 has the functions of both activator and repressor.^78^ Gli proteins are the key transcription factors in bone development. For instance, endochondral ossification was severely inhibited in Gli1 and/or Gli2 knockout mice, while Gli3 deletion mice displayed a significant increase in osteogenesis.^79^ Additionally, the SMO agonist SAG remarkably induced the expression of ALP and BSP (bone sialoprotein) in Gli1-overexpressed C3H10T1/2 cells. However, when Gli1 was silenced, the induction effect of SAG on ALP and BSP was correspondingly impaired.^79^ Overexpression of Gli2 not only induced the expression of ALP and OCN but also accelerated calcium deposition in MSCs and primary osteoblasts.^80^ These previous studies suggest that Gli1 and Gli2 may be positive regulators of osteogenic differentiation, and Gli3 may be a negative modulator. Furthermore, our recent study has shown that BMP9 elevated the mRNA levels of Gli1 and Gli2 in C3H10T1/2 cells,^56^ indicating that Gli1 and Gli2 may also be downstream targets of BMP9. In the present study, we confirmed that GANT-61, an inhibitor of Gli1 and Gli2, reversed the enhancing effect of Shh on BMP9-induced osteogenic differentiation of MSCs. These results suggest that Gli1 and Gli2 may act as essential mediators in the stimulatory effect of Shh on BMP9-induced osteogenic differentiation of MSCs.

In summary, our results indicate that Shh markedly enhances BMP9-induced osteogenic dedifferentiation of MSCs, and accelerates BMP9-promoted bone defect healing in rats. Therefore, the combination of BMP9 and Shh may hold great promise as a promising MSC-based therapy for the treatment of fracture nonunion, delayed fracture healing, and bone defects.

## Ethics declaration

The Animal Ethics Committee of Chongqing Medical University approved all experiments involving animals.

## CRediT authorship contribution statement

Xiaoji Luo and Jinyong Luo designed and supervised this investigation. Lulu Zhang and Caixia Ji conducted all the experiments. Lulu Zhang wrote the manuscript. Jinyong Luo revised the manuscript. Ziyun Li provided support in developing a standard protocol and establishing a subcutaneous MSC implantation model. Habu Jiwa and Zhou Xie assisted in establishing the rat cranial defect model. Lulu Zhang and Caixia Ji prepared and analyzed the experimental data.

## Funding

This study was supported by the Chongqing Young and Middle-aged Medical High-end Talent Project (China) (No. 2019GDRC001), Chongqing Talent Innovation and Entrepreneurship Leader Project (China) (No. cstc2022ycjh-bgzxm0103), the funding from Deyang Science and Technology Foundation (Sichuan, China) (No. 2021SZZ060), and the Program for Youth Innovation in Future Medicine of Chongqing Medical University (No. W0086).

## Conflict of interests

The authors declared no competing interests.
